# Interactive effects of light and snail herbivory rather than nutrient loading determine early establishment of submerged macrophytes

**DOI:** 10.1002/ece3.9070

**Published:** 2022-07-04

**Authors:** Mingjun Feng, Peiyu Zhang, Haowu Cheng, Thijs Frenken, Jun Xu, Min Zhang

**Affiliations:** ^1^ College of Fisheries Huazhong Agricultural University, Engineering Research Center of Green development for Conventional Aquatic Biological Industry in the Yangtze River Economic Belt, Ministry of Education, Hubei Provincial Engineering Laboratory for Pond Aquaculture Wuhan China; ^2^ Institute of Hydrobiology Chinese Academy of Sciences Wuhan China; ^3^ Cluster Nature and Society HAS University of Applied Sciences ’s‐Hertogenbosch the Netherlands; ^4^ Department of Aquatic Ecology Netherlands Institute of Ecology (NIOO‐KNAW) Wageningen the Netherlands

**Keywords:** aquatic plant, eutrophication, multiple stressors, periphyton, shading, stoichiometry

## Abstract

Submerged macrophytes play a key role in maintaining a clear‐water phase and promoting biodiversity in shallow aquatic ecosystems. Since their abundance has declined globally due to anthropogenic activities, it is important to include them in aquatic ecosystem restoration programs. Macrophytes establishment in early spring is crucial for the subsequent growth of other warm‐adapted macrophytes. However, factors affecting this early establishment of submerged macrophytes have not been fully explored yet. Here, we conducted an outdoor experiment from winter to early spring using the submerged macrophytes *Potamogeton crispus* and *Vallisneria spinulosa* to study the effects of shading, nutrient loading, snail herbivory (*Radix swinhoei*), and their interactions on the early growth and stoichiometric characteristics of macrophytes. The results show that the effects strongly depend on macrophyte species. Biomass and number of shoots of *P. crispus* decreased, and internode length increased during low light conditions, but were not affected by nutrient loading. *P. crispus* shoot biomass and number showed hump‐shaped responses to increased snail biomass under full light. In contrast, the biomass of the plant linearly decreased with snail biomass under low light. This indicates an interaction of light with snail herbivory. Since snails prefer grazing on periphyton over macrophytes, a low density of snails promoted growth of *P. crispus* by removing periphyton competition, while herbivory on the macrophyte increased during a high density of snails. The growth of *V. spinulosa* was not affected by any of the factors, probably because of growth limitation by low temperature. Our study demonstrates that the interaction of light with snail herbivory may affect establishment and growth of submerged macrophytes in early spring. Macrophyte restoration projects may thus benefit from lowering water levels to increase light availability and making smart use of cold‐adapted herbivores to reduce light competition with periphyton.

## Graphical abstract

Our study demonstrates that the interactions of light with snail herbivory, but not nutrient loading, may affect establishment and growth of submerged macrophytes in early spring. Our study thus has implications for recovery of submerged macrophytes in deteriorative water bodies.

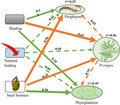

## 1 INTRODUCTION

Submerged macrophytes play vital roles in shallow aquatic ecosystems as they provide multiple key functions and ecosystem services, such as maintaining a clear water phase, providing habitat and shelter, producing food for animals, and by facilitating biodiversity (Carpenter & Lodge, 1986; O'Hare et al., 2017; Thomaz, 2021). Unfortunately, due to increased anthropogenic activities, the abundance and diversity of submerged macrophytes have declined in many shallow freshwater ecosystems worldwide (Sand‐Jensen et al., 2000; Zhang et al., 2017). Loss of submerged macrophytes also means the loss of a natural feedback loop that facilitates a clear‐water phase, and may result in the shift into an alternative stable state typically consisting of phytoplankton dominance and thus more turbid conditions. Consecutively, many functions and ecosystem services of aquatic ecosystems may be lost associated with this alternative turbid stable state (Hilt et al., 2017; Janssen et al., 2021; Phillips et al., 2016).

The recovery of a submerged macrophyte vegetation is one of the most important goals of lake ecological restoration (Hilt et al., 2018). During natural conditions, the recovery of submerged macrophytes from the turbid state should undergo different stages that may take several decades to complete (Hilt et al., 2018). The re‐occurrence of a spring clear‐water phase that can be exploited by a few macrophyte species (mainly pondweeds), after which a more diverse and abundant submerged macrophyte community can establish that stabilizes a clear‐water state for the rest of growing season. In recent years, more and more knowledge has been gathered on directly transplanting macrophytes in deteriorated water bodies to speed up re‐establishment and recovery of a macrophyte community, which may instead be achieved within 1 or 2 years (Li et al., 2021; Liu et al., 2018). However, there is little to no knowledge on which factors govern the success of re‐establishment of submerged macrophytes during and after restoration projects relying on transplantation of plants. Several abiotic factors may affect the growth of submerged macrophytes, such as light, temperature, and nutrients (Bornette & Puijalon, 2011). In addition, several biotic factors such as herbivory, bioturbation, and effects of trophic cascades, may reduce the growth of submerged macrophytes (Bakker et al., 2016; Hilt et al., 2018; Phillips et al., 2016; Zhi et al., 2020).

The underwater light environment is considered to be one of the most decisive factors affecting the distribution and growth of submerged plants (Middelboe & Markager, 1997). Shading by, for instance, suspended particulate matter, dissolved organic matter, and algae (phytoplankton and periphyton), may negatively affect the germination (Going et al., 2008; Havens et al., 2004) and growth (Bornette & Puijalon, 2011; Philbrick & Les, 1993) of submerged plants. The growth of submerged macrophytes is also (in)directly affected by nutrient loading (nitrogen, N and phosphorus, P). Generally, moderate nutrient loading would enhance the growth of macrophytes (Bakker et al., 2010; Cronin & Lodge, 2003; Ozimek et al., 1993). During eutrophic conditions, phytoplankton and periphyton growth is promoted, which can substantially reduce the growth of submerged macrophytes via increased light competition (Zhang et al., 2020). Furthermore, changes in light availability and nutrient loading may modify plant nutrient content, the carbon (C):nutrient [nitrogen (N) and phosphorus (P)] composition, and growth rates (Gu et al., 2018; Velthuis et al., 2017; Zhang et al., 2020). However, during winter, when temperatures are low, algae growth may be temperature limited (Edwards et al., 2016), and eutrophication may thus have less impact during these early stages of submerged macrophyte establishment.

Herbivory is also considered an important biological factor affecting the growth of submerged plants (Bakker et al., 2016). Previous studies show potential for complex relationships between snails, periphyton, and macrophytes (Bronmark, 1989; Jones et al., 1999; Underwood et al., 1992). Generally, periphyton is more palatable and the preferred food for snails over macrophytes (Bronmark, 1989; Guo et al., 2021; Koleszár et al., 2021; Mormul et al., 2010). At low density, snails can promote submerged macrophyte growth by removing periphyton from the surface of macrophytes and thus reducing the competition for light and nutrients with algae (Cao et al., 2014; Jones & Sayer, 2003; Underwood et al., 1992). At high densities, however, snails can inhibit macrophyte growth directly by grazing on the plant (Bronmark, 1990; Li, Liu, & Gu, 2009; Zhi et al., 2020).

Herbivory may also interact with nutrient loading to affect macrophyte and algal growth. During increased nutrient loading, the quality, namely lower C:nutrient ratios, may make primary producers more palatable to herbivores (Bakker et al., 2016; Liu et al., 2021). Similarly, herbivory may also interact with light, as reduced light may lower C:nutrient ratios of plants and algae also increasing palatability and nutritional quality of plants (Cronin & Lodge, 2003). Thus, during both increased nutrient loading and low light conditions, the negative effects of snails on macrophyte growth are expected to increase.

To test the effects of light, nutrient loading, and snail herbivory, and their interaction, on the growth and stoichiometry of submerged macrophytes, we performed an outdoor mesocosm experiment from winter to early spring using the two common rooted submerged vascular aquatic plants *Potamogeton crispus* and *Vallisneria spinulosa*. We hypothesize that (1) the growth of macrophytes will be limited by low light intensity, and will further decrease with increasing snail biomass. (2) Under high light, low snail biomass will promote macrophyte growth due to food preference on periphyton, releasing macrophytes from competition for light with periphyton, and high snail biomass will inhibit macrophyte growth, because snails will start feeding on macrophytes as a result of food limitation. (3) Nutrient loading will inhibit growth of submerged macrophytes, particularly under high snail biomass and low light conditions, as periphyton growth is stimulated and nutrient loading will increase quality of macrophytes for snails, namely higher nutrient contents and lower C:nutrient ratios.

## 2 MATERIALS AND METHODS

### 2.1 Study species

*Potamogeton crispus* (curly‐leaf pondweed) is native to Eurasia and invasive in North and South America and New Zealand. It is a cold‐adapted macrophyte with an optimum growth temperature in the range 10–20°C (Ren et al., 1997; Tobiessen & Snow, 1984). Turions of the plant germinate in fall, overwinter with little growth, and subsequently grow fast during early spring after which their growth subsides and they eventually die and senesce in summer (Catling & Dobson, 1985; Tobiessen & Snow, 1984; Zhang et al., 2022). *Vallisneria spinulosa* is endemic to China, mostly occurring in the middle and lower reaches of the Yangtze River (Wang, Song, et al., 2010), where it often coexists with *P. crispus*. *V. spinulosa* is a warm‐adapted species with an optimum temperature for germination at approximately 20°C (Wang et al., 2021). *Potamogeton crispus* turions and seedlings of *V. spinulosa* were obtained from another nearby lake, Lake Honghu (29°51′N, 113°20′ E), 2 days before the start of the experiment. *P. crispus* turions were stored in the dark at 4°C, and seedlings of *V. spinulosa* were kept fresh in a large tank (around 1000 L) with tap water.

The herbivore used in this experiment is *Radix swinhoei* (big‐ear radix), a pulmonated freshwater snail widely distributed throughout Asia, and found in a diverse set of habitats, including lakes, ponds, streams, rivers, and rice fields (Ziu et al., 1979). *Radix swinhoei* is a generalist grazer which feeds on both periphyton and macrophytes (Li, Liu, Hu, & Yang, 2009; Xiong et al., 2010). *Radix swinhoei* snails were collected in a pond in Huazhong Agricultural University Fisheries College Base in Wuhan City, Central China (30°29′N; 114°22′ E). All snails were collected by hands or fish nets, temporally stored in a bucket, sorted and evenly distributed to the selected containers at the first day of experiment.

### 2.2 Experimental design

Forty square polyethylene containers (around 100 L, 55 × 45 × 40 cm, l × w × h) were used as mesocosms and exposed to eight experimental treatments in a full factorial design (a control and three factors: shading, nutrient loading, and herbivory, each with two levels), with five replicates for each treatment (Figure S1), assigned randomly to the containers. The two selected light intensity levels were full sunlight (S0, high light intensity) and 25% sunlight (S1, low light intensity with shading). The two nutrient loading treatments consisted of nutrient loading (E1) and no external nutrient loading (E0) to the water column. Two snail herbivory treatments, one with (H1) and one without (H0) extra snails were added. The experiment lasted for 58 days, from January 26 to March 18, 2021. Tap water was added bi‐weekly to the containers to compensate for evaporation, however, the amount of water added to each container was little due to low evaporation during winter.

### 2.3 Experimental setup

The mesocosm is situated at Huazhong Agricultural University in Wuhan City, Central China (30°29′N, 114°22′ E). Ten centimeters of sediments were added which were collected from the top few cm of sediments in Lake Liangzi (N 30°11′3″, E 114°37′59″; sediment TN, 5.5 ± 0.4 mg g^−1^ and TP, 0.42 ± 0.08 mg g^−1^, dry weight, *n* = 5), and were thoroughly mixed in a clean container before transferring it into the experimental containers. On top of this, a layer 35 cm of tap water was added (TN, 2.438 ± 0.249 mg L^−1^; TP, 0.022 ± 0.006 mg L^−1^, *n* = 5). Each container received 12 *V. spinulosa* (shoot length:12.1 ± 2.0 cm, mean ± SD, *n* = 480) and 16 *P. crispus* turions (length:5.0 ± 0.5 cm, mean ± SD, *n* = 640) which were planted evenly distributed within each container.

The treatment with shading of 75% of the incoming sunlight (S1) was achieved by covering the containers with one layer of black sun‐shading net. The monthly means of daily radiation is 6.9 MJ m^−2^ day^−1^ in January in this area (Zhou et al., 2017). The nutrient solution used for the nutrient loading treatment (E1) was made by dissolving NaNO_3_ and KH_2_PO_4_ salts in demineralized water. Nutrients were added once a month to these E1 containers, simulating a high‐level nutrient loading of 2 mg L^−1^ N and 0.2 mg L^−1^ P, a eutrophic state, in the range of concentration and ratio of earlier experiments by Coppens et al. (2016) and Jeppesen et al. (2007). For the herbivory treatment, adult snails (*n* = 5, size ranged from 1 to 3 cm) were added to each herbivory treatment container (H1), a density of snails similar to natural lakes around the Yangtze River (Wang, Pan, et al., 2010), and no extra snails were added to the rest of the containers (H0). Due to snail mortality and reproduction, and some snails even emerged in the treatments without adding snails (H0), probably recovered from the sediment or hatched from eggs attached to the leaves of *V. spinulosa*, snail biomass changed largely over time in the treatments, snail biomass changed largely over time in the treatments. We thus used the final snail biomass achieved as a continuous variable to predict the response parameters in our experiment.

### 2.4 Data collection

Water quality parameters were measured six times during the experimental period, on day 0, 27, 37, 44, 51, and 58. Conductivity, temperature, and dissolved oxygen concentration (DO) were measured using a HACH portable multi‐parameter meter (HQ40d, HACH, USA). Total nitrogen (TN) and total phosphorus (TP) were determined using spectrophotometry after digestion with K_2_S_2_O_8_ in an autoclave (120°C, 1.1 kg cm^−2^) for 30 min, measuring at wavelengths 220 and 275 nm for TN, and 700 nm for TP (UV‐2800, Unico, China, GB 11894–89, GB 11893–89). At the end of the experiment, water was sampled from each experimental container and filtered onto GF/C filters to analyze chlorophyll a (Chl a) content as an indicator of phytoplankton biomass. Periphyton Chl a content (μg Chl a per cm^2^ area) was determined by brushing off a 9 cm^2^ (3 × 3 cm) area in the middle of one side wall after draining the water, filtering the brushed water through Whatman GF/C filters. Chl a content was determined spectrophotometrically after ethanol extraction (HJ 897–2017) (Chinese National Standards, 1996). Snails in each container were collected manually and wet weight was determined. Ten *P. crispus* were randomly selected in each container to measure internode length. All macrophytes were harvested with shoot and root separately cleaned and blotted dry to measure wet weight, after which they were dried in an oven at 60°C for 48 h. Dried plant samples were ground individually in a 2 ml tube on a mill (MiniBeadbeater‐16, Biospec Products, USA). Plant total C and N were determined on an elemental NC analyzer (Flash EA 1112, CE Instruments, Italy). P content was determined by incinerating and digesting the organic P with K_2_S_2_O_8_ (same to water TP analysis), and then measuring the dissolved phosphate concentration on a spectrophotometer (Cleverchem380, DeChem‐Tech., Germany).

### 2.5 Data analysis

Multiple generalized linear models (GLMs), using R package *lme4* with the “glm” function (Bates et al., 2015), were employed to analyze effects of light intensity, nutrient loading, snail biomass, and their interactions on primary producers (including *P. crispus* shoot biomass, shoot number and internode length, *V. spinulosa* total biomass, periphyton Chl a and phytoplankton Chl a content, C, N, P content and their ratios). Light intensity and nutrient loading were treated as categorical variables, and snail biomass as a continuous variable. Variance inflation factors (VIF) were calculated to test for collinearity problems that might arise from including interaction terms in the same model, and no collinearity problems were detected in all the models at the criteria of VIF <5 (Sheather, 2009). Due to the nonlinear response of *P. crispus* shoot biomass and shoot number to snail biomass under full light, the quadratic of snail biomass (poly function) was used as a predicted variable to analyze these effects, and the nonlinear responses were further confirmed by generalized additive models using “gam” function from R package *mgcv* (Wood, 2006) (Table S1). Since no significant effects of nutrient loading were observed on the growth of macrophytes and periphyton (Table 1), nutrient loading and the interaction with other factors were excluded and analyzed again in the GLMs (Table S2) and plotted. Multiple linear mixed‐effects models were applied to test effects of the treatments on water temperature, dissolved oxygen, conductivity, TN, and TP, with sampling date as a random factor. Estimated marginal means were compared after each linear model test to compare the difference between the treatments, using package *emmeans* (Lenth et al., 2022). QQplot and residual plot were used to visually assess the normality of data. If the data were not normally distributed, data were transformed (data transformation is added in Tables 1 and S2). One sample of *P. crispus* (in the E1S1 treatment) was missing after harvesting. This resulted in 39 *P. crispus* samples being available for the analysis of the three plant elemental compositions (plant C, N, and P content), and three plant stoichiometric traits (C: N, C:P, and N: P ratios).

**TABLE 1:** Effects of shading, herbivory, nutrient loading, and their interactions on the growth of primary producers, macrophyte elemental composition, and stoichiometry

Response		Shading	S	Herbivory	H	Eutrophication	E	Shading* Herbivory	C	Shading* Eutrophication	C	Herbivory* Eutrophication	C	Shading*Herbivory* Eutrophication
Primary producer	*P. crispus* biomass	**<0.001**	−					**0.008**	±					
	*P. crispus* Shoot number	**<0.001**	−											
	*P. crispus* internode length	**<0.001**	+	**0.032**	−									
	*V. spinulosa* biomass													
	log (Periphyton)			**<0.001**	−									
	log (Phytoplankton)	**<0.001**	+	**0.004**	+									**0.038**
*P. crispus* nutrient content	Carbon			**0.049**	+			**0.027**	±					
	Nitrogen													
	Phosphorus	**0.013**	−											**0.036**
*P. crispus* stoichiometry	C:N ratio													
	C:P ratio	**0.009**	+									**0.023**	±	**0.034**
	N:P ratio	**0.03**	+									**0.014**	±	

*Note*: Effects were analyzed by generalized linear models. Data transformation to meet model requirements is indicated. Due to the nonlinear response of *P. crispus* shoot biomass to snail biomass, the quadratic of snail biomass is used as a predicted variable in the models. For the manipulated factors shading (S), herbivory (H), and nutrient loading (E), main effects are classified directionally as positive (+) or negative (−) based on the response direction of manipulated versus control levels. Combined (C) two‐way interactions with herbivory are also classified directionally (+ or − effect of herbivory) and as stronger (>), weaker (<), or different (±) effects in the presence of the second stressor. “log” indicates the data are natural log transformed, respectively. Bold numbers indicate *p* < .05 and empty cells are not significant.

A structural equation model (SEM) was constructed to summarize the effects of shading, snail biomass, and nutrient loading on biomass of the macrophytes. This allowed assessing the complete graphical network of the interactions and relationships, with the directions of paths in the SEM diagram indicating causal influences (Rosseel, 2012). Quadratic of snail biomass was used to predict the response of *P. crispus* biomass. Three indices of model fit were used with conventional significance thresholds to assess the overall fit of the SEM, with the χ^2^ *p*‐value (*p* > .05), the standardized root mean squared residual (SRMR ≤0.08), and the comparative fit index (CFI ≥0.95) (Hu & Bentler, 1999). All SEM procedures were conducted with the *lavaan* (version 0.6‐3) package in R (Rosseel, 2012). All the analyses were performed using R software (ver. 4.0.1; R Core Team, 2021).

## 3 RESULTS

### 3.1 Water quality parameters

Shading reduced water temperature (S0, 18.12 ± 0.60°C; S1, 16.38 ± 0.50°C) and DO concentration (S0, 11.42 ± 0.18 mg L^−1^; S1, 7.85 ± 0.24 mg L^−1^), but increased conductivity (S0, 371.18 ± 3.77 μs cm^−1^; S1, 387.33 ± 3.16 μs cm^−1^), TN (S0, 1.80 ± 0.07 mg L^−1^; S1, 2.63 ± 0.07 mg L^−1^), and TP concentration (S0, 0.033 ± 0.01 mg L^−1^; S1, 0.045 ± 0.01 mg L^−1^) (Tables S3 and S4). TN concentration and conductivity were found to increase with snail biomass (Tables S3 and S4). Nutrient loading treatment increased conductivity. Shading, nutrient loading, and snail biomass also interactively affected DO concentration and conductivity (Table S3 and S4).

### 3.2 Effect of treatments on growth of primary producers

**FIGURE 1:**
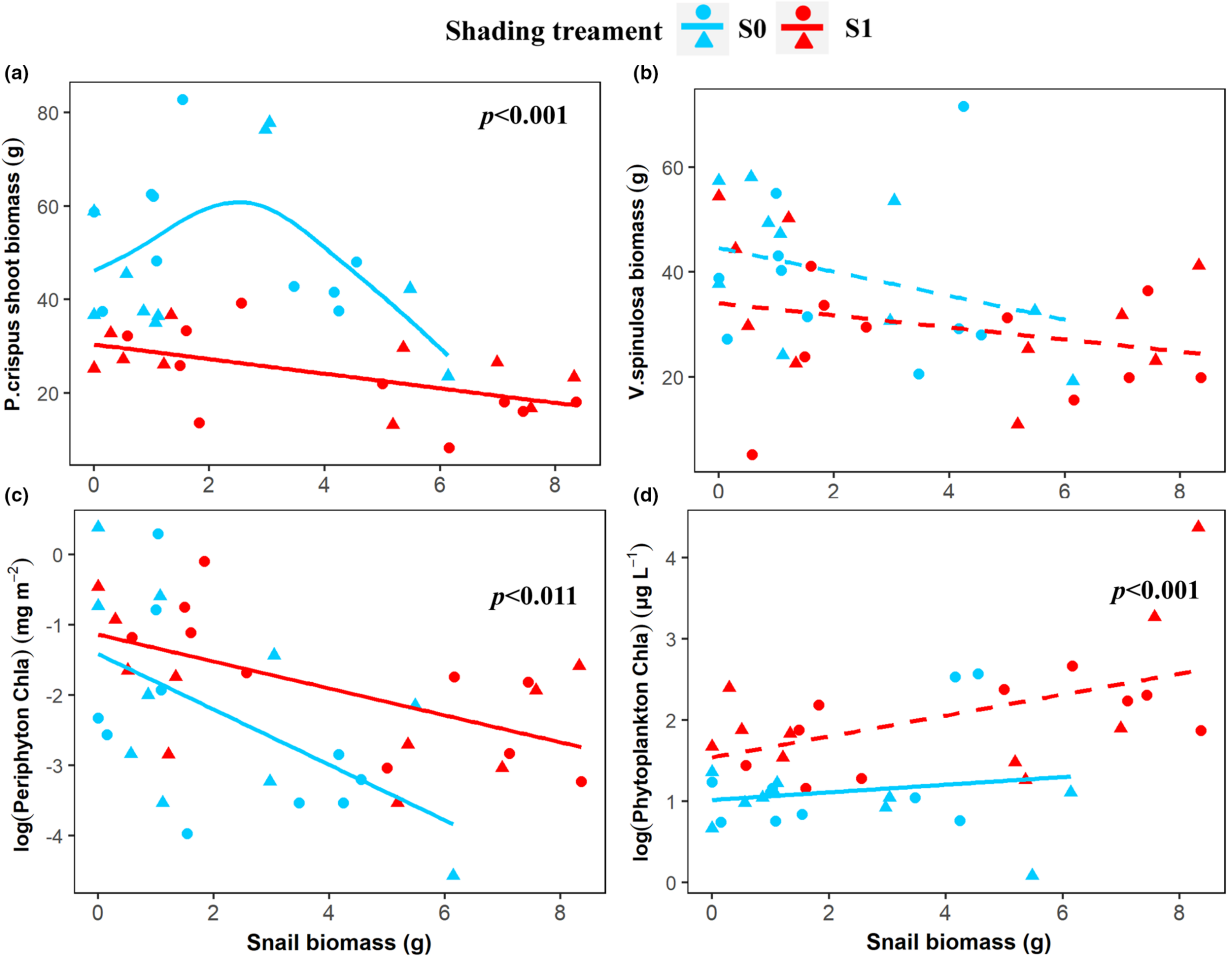
Effects of shading and snail biomass on primary producers. *P. crispus* shoot biomass (a), *V. spinulosa* biomass (b), periphyton chl a content (c), and phytoplankton chl a content (d). S0 indicates full light, S1 indicates low light intensity under shading. Lines are fitted by gam functions in package ggplot2, and a solid line indicates *p* < .05, and a dashed line indicates *p* > .05. Dot symbol indicates nutrient loading, and triangles indicate no additional nutrients were added. *p* values in the panel indicate a significant effect of shading on the mean of the response variable

**FIGURE 2:**
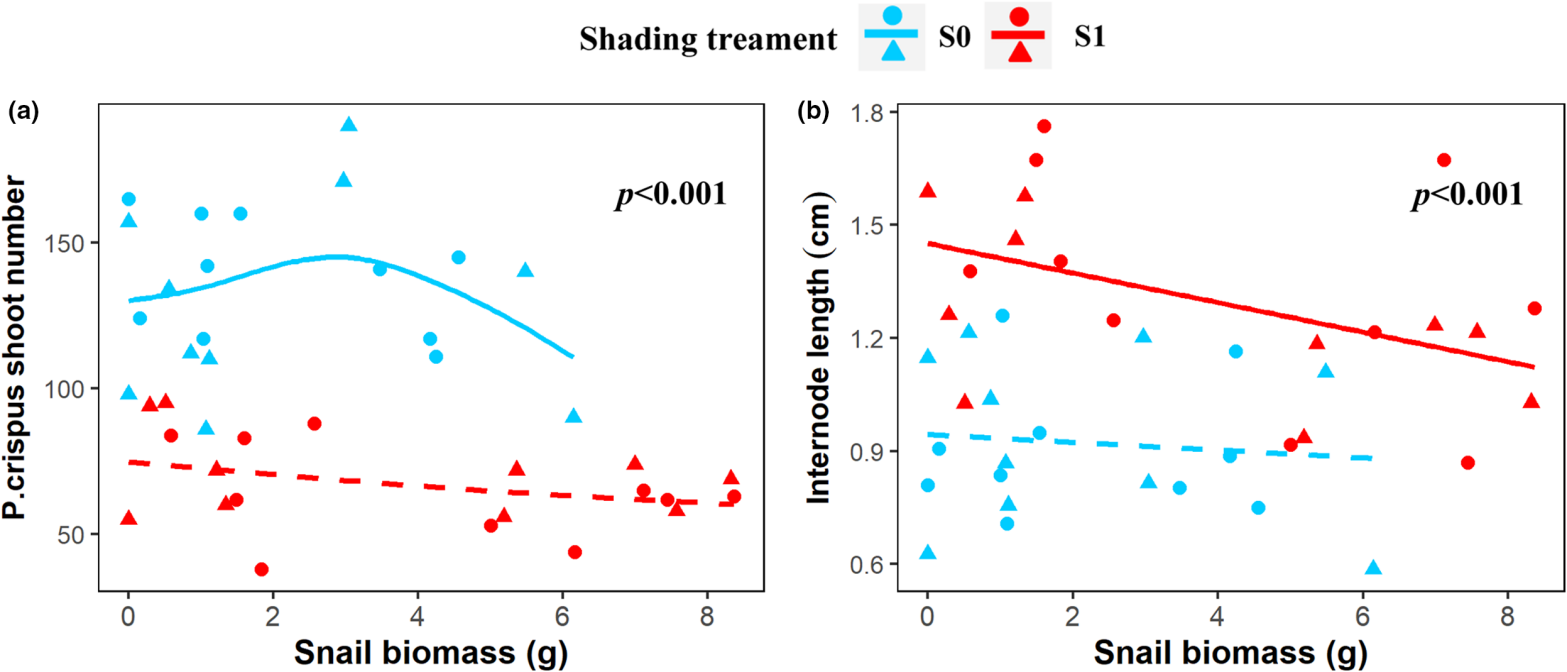
Effects of shading and snail biomass on shoot number and internode length of *P. crispus*. Shoot number (a) and internode length (b). S0 indicates full light, S1 indicates low light intensity under shading. Lines are fitted by gam functions in package ggplot2, and a solid line indicates *p* < .05, and a dashed line indicates *p* > .05. Dot symbol indicates nutrient loading, and triangles indicate no additional nutrients were added. *p* values in the panel indicate a significant effect of shading on the mean of the response variable

Nutrient loading did not affect the biomass of both submerged plants (Table 1). Shading decreased *P. crispus* shoot biomass and shoot number, but increased *P. crispus* internode length (Figures 1a and 2a,b). Shading and snail biomass interactively affected the shoot biomass of *P. crispus* (Table 1, Figure 1a). In the full light treatment, shoot biomass and number of *P. crispus* showed a humped‐shape response to snail biomass, but shoot biomass declined with snail biomass in the shading treatment (Figures 1a and 2a). No treatment effects were observed for the biomass of V. spinulosa (Table 1 and Figure 1b). Nutrient loading did not affect periphyton biomass. Shading significantly increased biomass of periphyton and phytoplankton. Periphyton biomass decreased with snail biomass under both full light and shaded conditions, while phytoplankton increased with increasing snail biomass only during shading (Table 1 and Figures 1c, d).

### 3.3 Effect of treatments on stoichiometry of macrophyte

**FIGURE 3:**
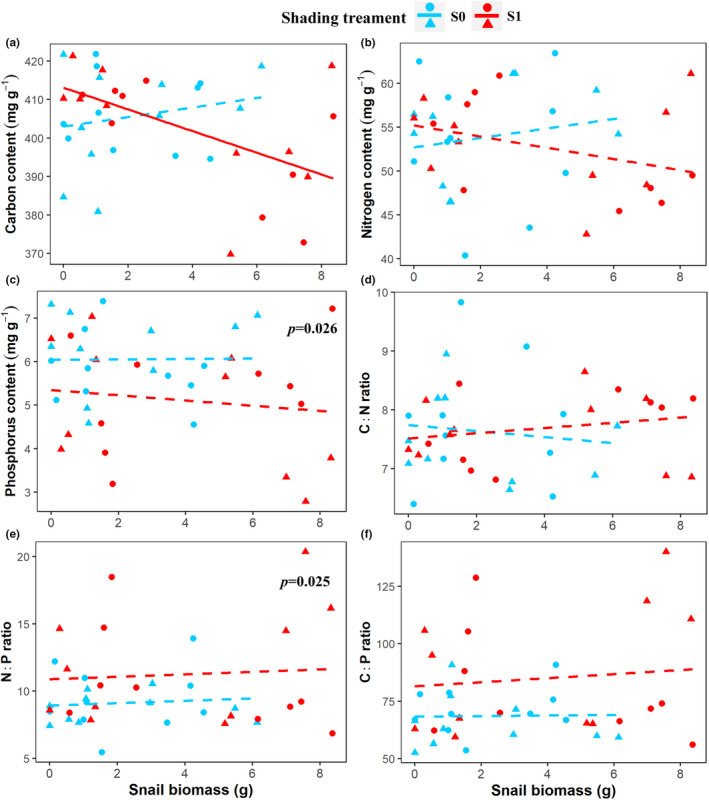
Effects of shading, snail biomass, and nutrient loading on *P. crispus* elemental composition (C, N, and P contents) and stoichiometry (C:N, C:P, and N:P ratio). *P. crispus* C content (a), N content (b), P content (c), C:N ratio (d), C:P ratio (e), and N:P ratio (f). A solid line indicates *p* < .05, and dashed line indicates *p* > .05. Dots indicate nutrient loading, and triangle points indicate no nutrient loading. *p* values in the panel indicate significant difference in the means between the two light intensity treatment

No changes in N content and C: N ratio of *P. crispus* could be detected in any of the treatments (Table 1 and Figure 3b, d). The P content of *P. crispus* decreased and C:P ratio increased under shading (Table 1 and Figure 3c, f). Plant C content decreased with snail biomass during shaded conditions (Table 1 and Figure 3a). Nutrient loading and snail biomass interactively affected C:P and N:P ratios of *P. crispus*, and the two treatments together with shading affected the P content and C:P ratio of *P. crispus* (Table 1).

### 3.4 Structural equation model

**FIGURE 4:**
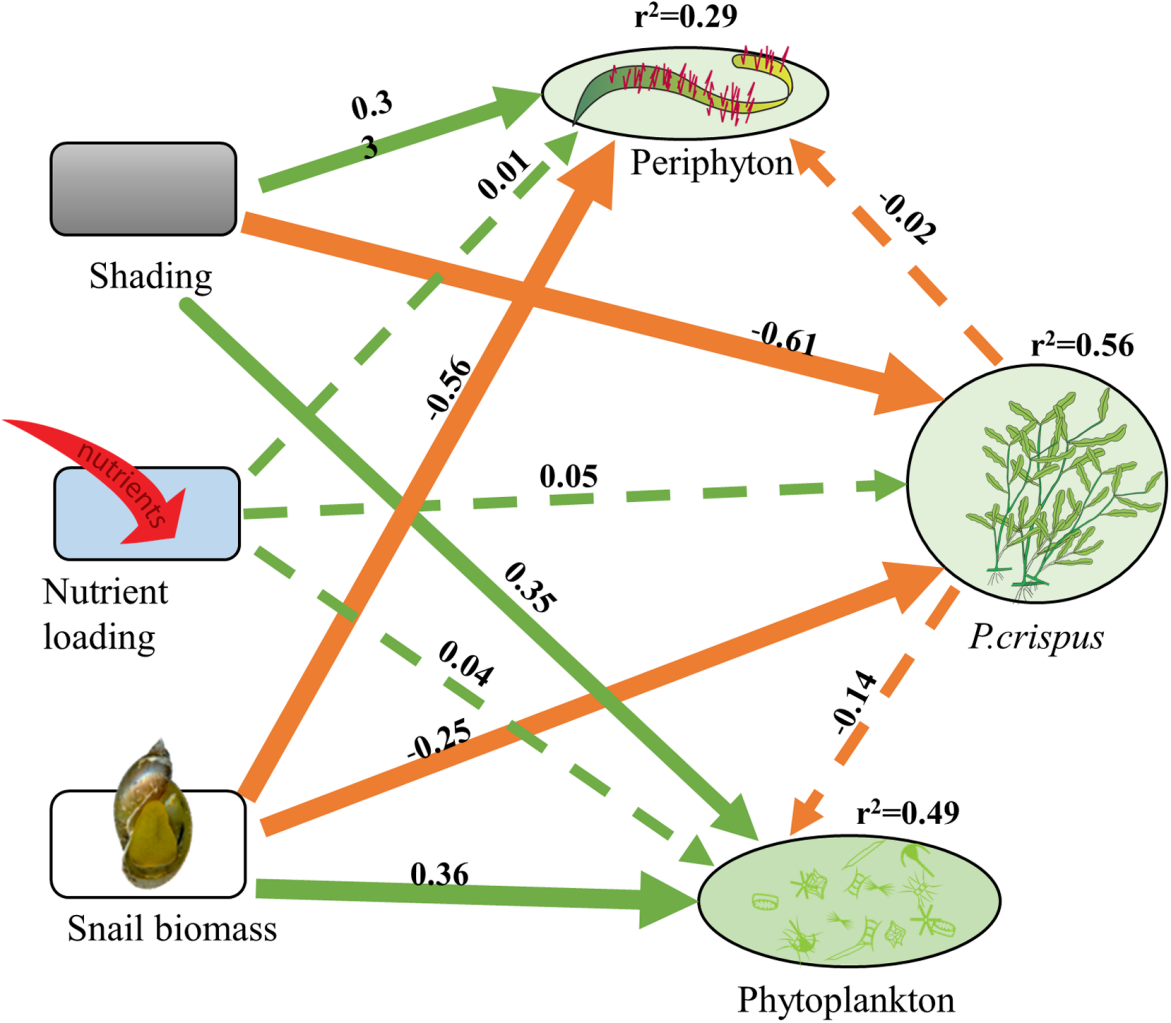
Structural equation model (SEM) of shading, nutrient loading, and snail biomass effects on the growth of primary producers. Exogenous variables are indicated by rounded rectangles, and endogenous variables are represented by ovals. Coefficients of determination (*r* ^2^) are shown for all endogenous variables. Numbers adjacent to arrows are standardized path coefficients and indicate the effects of the relationship. Positive and negative effects among variables are depicted by green and orange arrows, respectively, with arrow thicknesses proportional to the strength of the relationship. Significant pathways (*p* < .05) are represented by solid lines, otherwise by dashed lines. The model satisfied each of the three model fit criteria with significant χ^2^ of *p* = .239, standardized root mean squared residuals of 0.012, and comparative fit index values of 0.981

The structural equation model indicates that both shading and snails affect primary producer biomass (Figure 4). Shading and snail herbivory both negatively affected biomass of *P. crispus* (standardized path coefficient, SPC = −0.61 and − 0.25, respectively). Snail herbivory decreased (SPC = −0.56), and shading increased (SPC = 0.32) biomass of periphyton (*r* ^2^ = 0.29). Both shading (SPC = 0.35) and snail (SPC = 0.36) significantly increased the biomass of phytoplankton (*r* ^2^ = 0.49).

## 4 DISCUSSION

Light intensity, nutrient loading, and snail herbivory interactively affected the growth of submerged macrophytes, phytoplankton, and periphyton during the start of the growing season. Our experiment indicated that shading substantially reduced the total biomass and shoot number of *P. crispus* compared to full light conditions. *P. crispus* shoot biomass decreased with increasing snail herbivory under shaded conditions, which confirms the first hypothesis. Under full light conditions, *P. crispus* shoot biomass first increased and then decreased with increasing of snail biomass, which is in line with the second hypothesis. Additionally, shading promoted the growth of periphyton and phytoplankton, and periphyton biomass decreased with increased snail biomass. These interactions were furthermore confirmed by a structural equation model. However, no effects of nutrient loading and the interactions with light intensity nor snail herbivory on the growth of primary producers could be detected, thus rejecting the third hypothesis.

### 4.1 Effects of shading

Shading significantly reduced the shoot number and biomass, but increased the internode length of the submerged macrophyte *P. crispus*. Light is one of the primary factors determining photosynthetic rate of plants, and reducing light availability thus is expected to inhibit the growth of submerged macrophytes, which has indeed been demonstrated in a number of studies (Chou et al., 2022; Middelboe & Markager, 1997; Riis et al., 2012), and is in line with our findings. Shading, however, may also promote macrophyte elongation as a strategy to cope with light limitation as seen in this and previous studies (Chou et al., 2022; Riis et al., 2012). Light also indirectly changed growth conditions of macrophytes in this experiment, as the average water temperature was, for instance, almost 2°C lower in the shaded treatment as compared to the full light treatment. Increased temperature has been shown to promote the growth of macrophytes (Puche et al., 2018; Rojo et al., 2017; Zhang et al., 2016; Zhang et al., 2022), and thus in part may explain why *P. crispus* accumulated less biomass under the shaded colder conditions. No effects of shading could be detected on the growth of *V. spinulosa*, as the plant grew little in any of the treatments. *Vallisneria* species are warm‐adapted species, and the plant thus may have grown little due to the low temperature in our experiment (Bartleson et al., 2014; Zhang et al., 2021).

Although shading reduced growth of *P. crispus*, biomass buildup of the other primary producers was promoted. It could be expected that reduced light availability may decrease primary producer biomass (Edwards et al., 2016; Karlsson et al., 2009). However, in a naturally complex shallow aquatic ecosystem with macrophytes, periphyton, and phytoplankton present, macrophytes may be more sensitive to light limitation, as they cannot win competition for light from periphyton and phytoplankton (Scheffer, 2004; Yamamichi et al., 2018). Low light conditions may thus counterintuitively result in increased phytoplankton and periphyton biomass since they will be released from competition with macrophytes (Guan et al., 2020; Yamamichi et al., 2018). This could also explain why shading increased TN and TP concentrations in our study. The algal community may have also shifted toward species that prefer low light conditions (Schwaderer et al., 2011), thus maintaining growth rates and biomass (Mette et al., 2011). Shading reduced light intensity, as well as ultraviolet radiation, which might have direct effects on the organisms, and these effects are species‐specific (Rojo et al., 2019; Rubio et al., 2015). This might partly explain the different response of primary producers in our study.

### 4.2 Interactive effects of snail herbivory and shading

Shading and snail herbivory interactively affected the growth of *P. crispus*. Under full light conditions, low biomass of snails promoted the growth of *P. crispus*, while during high biomass of snails the macrophyte growth was inhibited. This may be a consequence of preferential feeding of snails on periphyton over macrophytes (Bronmark, 1989; Guo et al., 2021; Koleszár et al., 2021; Zhi et al., 2020). During low biomass of snails, there is plenty of periphyton available to graze, and herbivory in this case releases macrophytes from competitive pressure for light with periphyton, promoting the growth of macrophytes (Yang et al., 2020). In contrast, during high biomass of snails, periphyton food is depleted and snails will graze on macrophytes even under full light conditions, thus inhibiting their growth (Zhi et al., 2020). However, during shaded conditions, the preferential food availability (periphyton) to snails is limited, forcing snails to graze on macrophytes, thus we observed a continuous decline of *P. crispus* biomass with increasing of snail biomass.

### 4.3 Effects of nutrient loading

Increased nutrient loading was expected to increase growth of algae, and inhibit growth of macrophytes, as fast‐growing phytoplankton and periphyton may outcompete macrophytes for nutrient and subsequently light (Scheffer, 2004; Zhang et al., 2020). However, we did not find an effect of nutrient loading on the growth of macrophytes and periphyton, which is in accordance with previous study showing that moderate concentrations of nitrogen and phosphorus did not have a significant impact on the germination and seedling growth of *P. crispus* (Gao et al., 2005) and charophytes (Rodrigo et al., 2018). This may be a result of algal growth limitation by low temperatures, or due to other interactions with macrophytes such as, for instance, allelopathy (Hilt, 2006; Pakdel et al., 2013). *P. crispus* did not suffer from low temperature since it is a cold‐adapted species that can germinate below 10°C (Ren et al., 1997). Once temperatures rose it was already established and could quickly start growing in early spring, thus potentially outcompeting or partially suppressing growth of phytoplankton. An alternative explanation could be that in our experimental systems P may have limited growth of phytoplankton or periphyton, indicated by the relatively elevated TN:TP ratio in the water (N:P = 25.7 by atoms) (Klausmeier et al., 2004), but not impacting the macrophytes since they can access P from the sediment too. Though no significant direct impact of nutrient loading on the growth of macrophytes was detected, nutrient loading did interact with other treatments to affect P content, C:P, and N:P ratios of the plant. These changes in nutrient content and stoichiometry may have impacts on herbivory via changes in food quality and palatability (Bakker & Nolet, 2014; Frost et al., 2006).

### 4.4 Implications for lake restoration

To restore a clear‐water phase in shallow aquatic ecosystems, the re‐establishment of a *P. crispus* population during the end of winter and early spring can help recover other submerged macrophytes, as the growth of *P. crispus* can suppress the growth of algae and keep the water clear, improving conditions for the establishment of other macrophytes (Hilt et al., 2006; Hilt et al., 2018). As shown here, however, it is important to take light availability and herbivory into consideration, as these can significantly affect successful establishment of *P. crispus*, particularly with ongoing climate change that may enhance herbivory on macrophytes (Bakker et al., 2016). Lowering water level could be a good measure to increase light availability and thus increase *P. crispus* growth. Other measures to prevent herbivory can also be applied, for example, stocking of their predators or some parasites of the snail, and may be especially needed during high light conditions.

Furthermore, macrophytes with different thermal optima could be combined, as shown here where a low temperature prevented the growth of *V. spinulosa*. Since *P. crispus* does not tolerate high temperatures, it would die off during warmer conditions in summer, and it is thus essential to combine it with other macrophytes that germinate later and can handle warmer waters. In addition, with global climate change (IPCC, 2014), cold‐adapted species might suffer more from warming through advancing growth and senescence, and warming might further enhance the top‐down grazing effects of herbivores on macrophytes (Zhang et al., 2022). If no other macrophytes establish during the senescence of *P. crispus* the water may become dominated by phytoplankton and risks being shifted into a turbid state. This might further impair benthic food webs, a key pathway in shallow water bodies, which has largely been ignored (Puche et al., 2021; Vadeboncoeur et al., 2003).

## AUTHOR CONTRIBUTIONS

**Mingjun Feng:** Data curation (equal); investigation (lead); writing – original draft (lead); writing – review and editing (equal). **Peiyu Zhang:** Conceptualization (lead); formal analysis (equal); investigation (equal); writing – review and editing (equal). **Haowu Cheng:** Data curation (equal); investigation (equal); writing – review and editing (equal). **Frenken Thijs:** Data curation (equal); writing – review and editing (equal). **Min Zhang:** Conceptualization (equal); supervision (equal); writing – review and editing (equal). **Jun Xu:** Conceptualization (equal); supervision (equal); writing – review and editing (equal).

## CONFLICT OF INTEREST

The authors have no relevant financial or nonfinancial interests to disclose.

## Supporting information


Appendix S1

## Data Availability

Data are available at Dryad https://doi.org/10.5061/dryad.f4qrfj6zj.
