# Supplementary material for: Interactive effects of light and snail herbivory rather than nutrient loading determine early establishment of submerged macrophytes
Source: Ecol Evol. 2022 Jul 4;12(7):e9070. doi: 10.1002/ece3.9070 (PMC9251838; doi:10.1002/ece3.9070)
Supplement: Supplementary file 1 — Appendix S1 [file ECE3-12-e9070-s001.docx]

**Appendices**

The following materials are included in the appendices:

**Figure S1** Pictures of the experiment.

**Table S1** Effects of shading, herbivory, and their interactions on the growth of primary producers, macrophyte nutrient content and stoichiometry.

**Table S2** Effects of snail biomass on *P. crispus* biomass and shoot number under different light levels.

**Table S3** Averaged water quality parameters in each treatment during the experiment.

**Table S4** Effects of Shading, snail herbivory, nutrient loading and their interactions on water quality parameters.


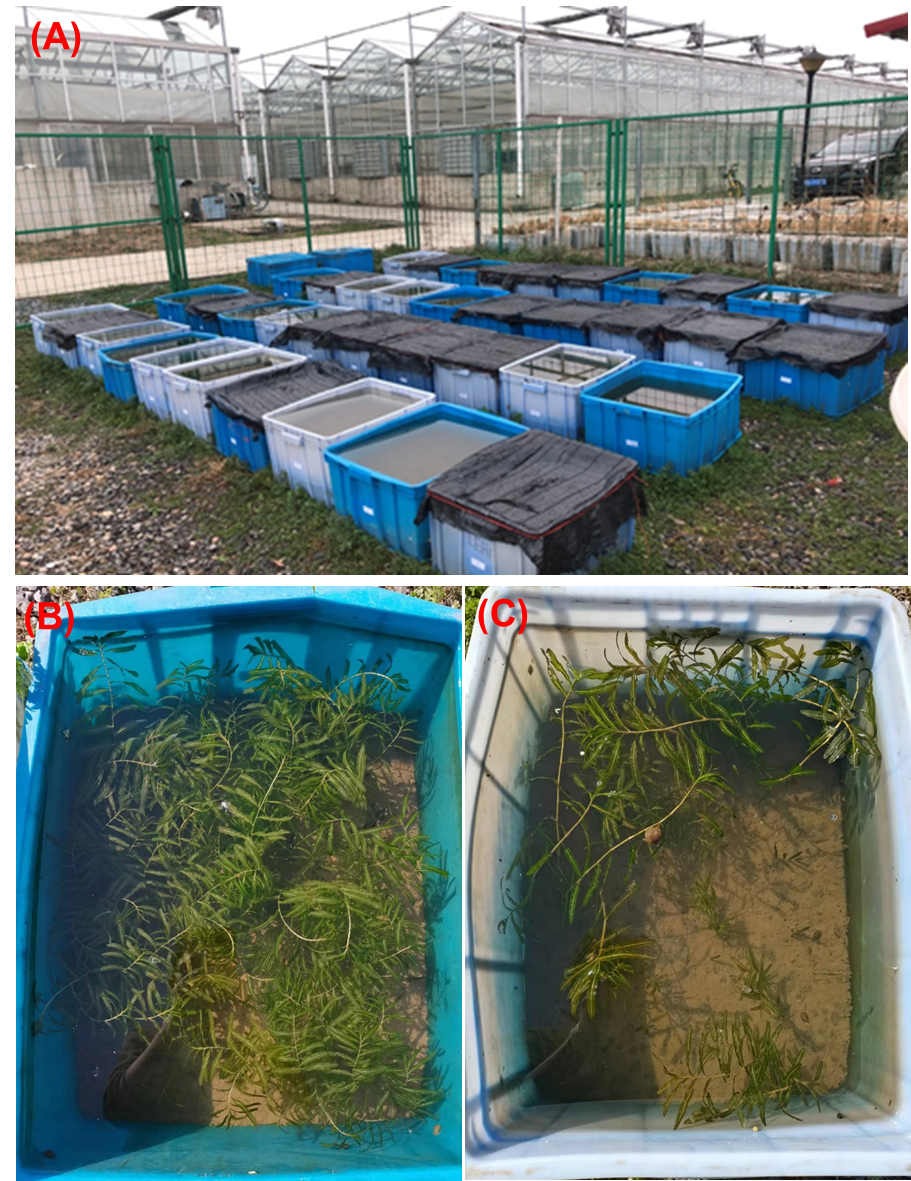
**Figure S1** Experiment photos. (A) Experiment layout, (B) Mesocosm with low snail biomass without shading, and (C) mesocosm with high snail biomass with shading.

**Table S1** Effects of snail biomass on *P. crispus* biomass and shoot number under different light levels, tested by generalized additive models. S0 indicates full light, S1 indicates low light intensity under shading.

| Response | Treatment | *F* | *p*-value |
| --- | --- | --- | --- |
| *P. crispus* biomass | S0 | 14.14 | <0.001 |
|  | S1 | 5.62 | 0.024 |
| *P. crispus* shoot number | S0 | 17.53 | <0.001 |
|  | S1 | 1.61 | 0.213 |

**Table S2** Effects of shading, herbivory, and their interactions on the growth of primary producers, macrophyte elemental composition and stoichiometry. Effects were analyzed by generalized linear models. Data transformation to meet model requirements is indicated. Due to the nonlinear response of P. crispus shoot biomass to snail biomass, the quadratic of snail biomass is used as a predicted variable in the models. For the manipulated factors shading (S) and herbivory (H), main effects are classified directionally as positive (+) or negative (−) based on the response direction of manipulated versus control levels. Combined (C) two- way interactions with herbivory are also classified directionally (+ or − effect of herbivory) and as stronger (>), weaker (<) or different (±) effects in the presence of the second stressor. “log” indicates the data are natural log transformed. Bold numbers indicate *p* < 0.05.

| Response |  | Shading | S | Herbivory | H | Shading*Herbivory | C |
| --- | --- | --- | --- | --- | --- | --- | --- |
| Primary producer | *P. crispus* biomass | **<0.001** | **－** | 0.054 |  | **0.018** | **±** |
|  | *P. crispus* Shoot number | **<0.001** | **－** | 0.722 |  | 0.813 |  |
|  | *P. crispus* internode length | **<0.001** | **+** | **0.031** | **－** | 0.360 |  |
|  | *V. spinulosa* biomass | 0.091 |  | 0.078 |  | 0.541 |  |
|  | log(Periphyton) | **0.018** | **+** | **<0.001** | **－** | 0.158 |  |
|  | log(Phytoplankton) | **<0.001** | **+** | **0.007** | **+** | 0.343 |  |
| *P. crispus* nutrient content | Carbon | 0.886 |  | **0.039** | **+** | **0.019** | **±** |
|  | Nitrogen | 0.765 |  | 0.440 |  | 0.159 |  |
|  | Phosphorus | **0.029** | **－** | 0.578 |  | 0.687 |  |
| *P. crispus* stoichiometry | C:N ratio | 0.928 |  | 0.743 |  | 0.382 |  |
|  | C:P ratio | **0.026** | **+** | 0.604 |  | 0.788 |  |
|  | N:P ratio | 0.069 |  | 0.657 |  | 0.996 |  |

**Table S3** Averaged water quality parameters in each treatment during the experiment. Mean ± SD.

| Treatments | Temperature | DO | Conductivity | TP | TN |
| --- | --- | --- | --- | --- | --- |
|  | (°C) | (mg L^-1^) | (µs cm^-1^) | (mg L^-1^) | (mg L^-1^) |
| Control | 18.06±6.64 | 11.54±1.80 | 362.80±38.66 | 0.039±0.04 | 1.60±0.85 |
| Herbivory | 18.06±6.57 | 11.27±2.06 | 370.43±47.72 | 0.025±0.02 | 1.81±0.77 |
| Eutrophication | 18.23±6.76 | 11.37±2.03 | 377.50±39.41 | 0.034±0.04 | 1.61±0.64 |
| Eutrophic* Herbivory | 18.15±6.66 | 11.48±2.18 | 374.00±39.33 | 0.035±0.02 | 2.20±0.70 |
| Shading | 16.43±5.53 | 7.97±2.17 | 379.67±34.19 | 0.046±0.04 | 2.50±0.76 |
| Shading* Herbivory | 16.36±5.52 | 7.99±3.10 | 385.47±37.14 | 0.048±0.04 | 2.72±0.65 |
| Shading* Eutrophication | 16.30±5.43 | 8.30±2.56 | 386.63±33.19 | 0.040±0.04 | 2.37±0.81 |
| Shading* Eutrophication* Herbivory | 16.41±5.63 | 7.12±2.64 | 397.53±32.98 | 0.046±0.04 | 2.93±0.82 |

**Table S4** Effects of Shading, snail herbivory, nutrient loading and their interactions on water quality parameters. The effects were analyzed by linear-mixed effect models. *P* values are presented. Bold numbers indicate *p* < 0.05.

| Treatments | Temperature | DO | Conductivity | TP | TN |
| --- | --- | --- | --- | --- | --- |
| Shading | **<0.001** | **<0.001** | **<0.001** | **0.005** | **<0.001** |
| Herbivory | 0.922 | 0.099 | **0.016** | 0.976 | **<0.001** |
| Eutrophication | 0.735 | 0.648 | **<0.001** | 0.813 | 0.266 |
| Shading* Herbivory | 0.83 | 0.71 | 0.677 | 0.215 | 0.301 |
| Shading* Eutrophication | 0.635 | 0.889 | 0.846 | 0.288 | 0.168 |
| Eutrophication* Herbivory | 0.879 | 0.289 | 0.657 | 0.44 | 0.193 |
| Shading* Eutrophication* Herbivory | 0.438 | **0.015** | **0.009** | 0.738 | 0.937 |
